# Effects of High-Speed Power Training on Muscle Performance and Braking Speed in Older Adults

**DOI:** 10.1155/2012/426278

**Published:** 2012-02-28

**Authors:** Stephen P. Sayers, Kyle Gibson

**Affiliations:** Department of Physical Therapy, University of Missouri, Columbia, MO 65211, USA

## Abstract

We examined whether high-speed power training (HSPT) improved muscle performance and braking speed using a driving simulator. 72 older adults (22 m, 50 f; age = 70.6 ± 7.3 yrs) were randomized to HSPT at 40% one-repetition maximum (1RM) (HSPT: *n* = 25; 3 sets of 12–14 repetitions), slow-speed strength training at 80%1RM (SSST: *n* = 25; 3 sets of 8–10 repetitions), or control (CON: *n* = 22; stretching) 3 times/week for 12 weeks. Leg press and knee extension peak power, peak power velocity, peak power force/torque, and braking speed were obtained at baseline and 12 weeks. HSPT increased peak power and peak power velocity across a range of external resistances (40–90% 1RM; *P* < 0.05) and improved braking speed (*P* < 0.05). Work was similar between groups, but perceived exertion was lower in HSPT (*P* < 0.05). Thus, the less strenuous HSPT exerted a broader training effect and improved braking speed compared to SSST.

## 1. Introduction

Resistance training is a commonly prescribed and broadly researched rehabilitative strategy for older adults to maintain or improve muscle strength and function. Resistance training interventions typically emphasize high-load, strengthening exercise; however, muscle power (force × velocity) has emerged as an important muscle performance characteristic in this population [1–7]. A key component of muscle power is the speed at which force is developed. Resistance training using high movement speeds and high external resistance [8] or high movement speeds and low external resistance [9–11] have demonstrated positive impact on both muscle power and some functional performance tests. A recent meta-analysis revealed that various forms of high-speed resistance training (i.e., power training) were more effective at improving muscle power with only a small impact on function compared to traditional slow-speed strength training [12].

In older adults, muscle power declines at up to twice the rate than muscle strength (3-4% versus 1-2%), mostly due to declines in velocity compared to force [13, 14]. Thus, interventions that potentially improve muscle power and the velocity component of power may be critical in this population, especially with regard to function. Different functional tasks, however, may require power with a greater velocity component or a greater force component depending on the nature of the specific task (e.g., moving the lower limb quickly to keep from falling versus slowly getting up from a chair); thus, different resistance training protocols may be able to deliver different aspects of power to transfer to functional task performance. We believe resistance training programs to improve velocity could have a significant impact on functional tasks related to safety in this population. For example, exercise that improves the ability to rapidly move the lower limb from the accelerator to the brake while driving would have significant public health implications because motor vehicle accidents are one of the leading causes of injury-related deaths in older adults [15]. One recent study has shown that ankle power training increased foot movement speed compared to control using a left and a right foot switch [16]. Because of the importance of rapidly braking an automobile, however, we believe it is critical to replicate both the equipment found in an automobile and the mechanics of the braking motion.

Few studies in older adults have focused on the key components of muscle power (velocity and force) and how these variables are impacted by different resistance training protocols. The purpose of this study was to examine the effect of high-speed power training on muscle power and its components in older men and women and how changes in those parameters with training impacted braking speed using a driving simulator. We hypothesized that high-speed power training would improve power and the velocity component of power at lower external training resistances and transfer successfully to improve braking speed compared to slow-speed strength training.

## 2. Materials and Methods

### 2.1. Participants

Eligible participants had to be between the age of 65–90 years, ambulatory with or without an assistive device (cane or 3 post walker only) and community dwelling. Exclusion criteria consisted of history of heart disease, severe visual impairment, presence of neurological disease, pulmonary disease requiring the use of oxygen, uncontrolled hypertension, hip fracture or lower extremity joint replacement in the past 6 months, and current participation in structured exercise. A study physician determined medical eligibility for all participants.

**Figure 1:**
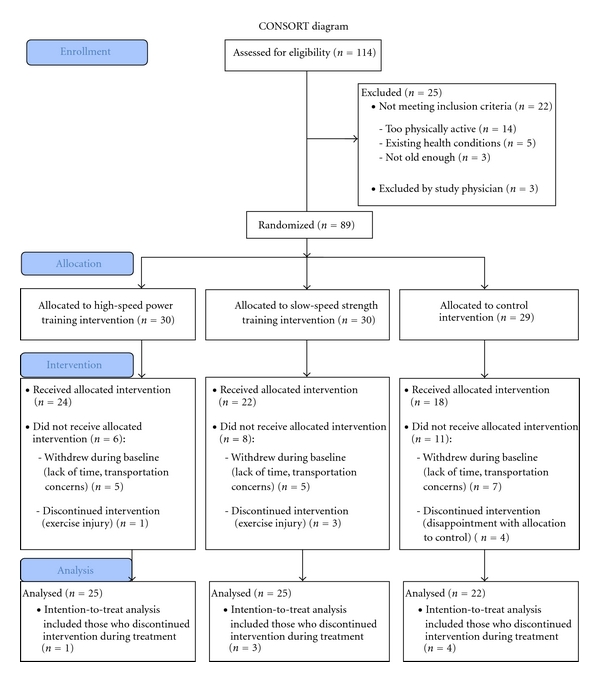
Overview of recruitment of study participants and randomization to study arms. HSPT = high-speed power training; SSST = slow-speed strength training; CON = Control.

One hundred and fourteen individuals were contacted to participate in the study. Eighty-nine individuals were randomized to one of three groups: high-speed power training (HSPT: *n* = 30), slow-speed strength training (SSST: *n* = 30), and control (CON: *n* = 29) (see CONSORT diagram Figure 1 for details). Seventeen participants dropped out during the baseline period before training had begun. Eight participants withdrew during the intervention, 1 in HSPT, 3 in SSST, and 4 in CON, three of which were study-related (HSPT (*n* = 1) and SSST (*n* = 2)). Using an intention-to-treat design, the data from 72 older men and women (HSPT: *n* = 25; STR: *n* = 25; CON: *n* = 22) were analyzed in this study. Subject characteristics are presented in Table 1. This project was approved by the University of Missouri Institutional Review Board and written consent was obtained from all participants.

### 2.2. Procedures

The study compared 12 weeks of explosive high-speed power training with traditional slow-speed strength training. Primary outcome measures included muscle performance: leg press and knee extension one-repetition maximum (1RM), muscle power across a range of external resistances (40–90% 1RM), and the corresponding velocity at peak power and force/torque at peak power. The primary measures were chosen to determine whether high-speed training would improve critical muscle performance variables (power and speed) necessary for function in older adults. For brevity only leg press measures are presented. A secondary outcome measure included high-speed function: braking speed using an automobile driving simulator. This secondary outcome measure was necessary to demonstrate whether changes in primary outcomes translated to actual functional performance. Tertiary outcome measures included ratings of perceived exertion (RPE) and total work performed during the training. These latter measures were included as an exploratory analysis of how individuals responded to the different exercise regimens and to ensure that the volume of exercise in both training groups was comparable, respectively.

Participants reported to the laboratory for 2 weeks of baseline measurements. On visit 1, body mass was recorded on a platform scale to the nearest 0.1 kg with the subject fully clothed. Height was measured to the nearest 0.5 cm with a scale stadiometer. Body mass index was calculated from these variables. Global cognitive function was assessed by the Mini-Mental State Examination [17], and the Geriatric Depression Scale was administered to assess depression over the previous week [18]. Number of falls in the past year and daily medications were assessed via questionnaire. On visit 2 and 3, muscle performance and functional measures were obtained. The following week, all muscle performance and functional measures were repeated to establish reliability. If baseline 1RM measurements deviated by more than 10% in repeated attempts, a third measure was obtained. At the end of baseline testing, participants were randomized to treatment. Following the 12-week resistance training intervention, posttraining muscle performance and functional measures were obtained.

### 2.3. Resistance Training Protocol

Volunteers randomized into HSPT and SSST exercised 3 times per week for 12 weeks using computer-interfaced Keiser a420 pneumatic leg press and seated knee extension resistance training equipment (Fresno, CA). For HSPT, each training session consisted of 3 sets of 12–14 repetitions at 40% 1RM. Participants performed an explosive movement at high speed during the concentric phase of each repetition, paused for one-second, and performed the eccentric portion of the contraction over 2-3 seconds. Volunteers randomized into SSST also exercised 3 times per week for 12 weeks with each training session consisting of 3 sets of 8–10 repetitions at 80% 1RM. Repetition number was higher in the HSPT group to more closely equate work performed between groups and to remain consistent with resistance training guidelines for exercise using lower external resistances [19]. The participants performed each movement at a slow velocity (2-3 s for concentric phase of the repetition), paused for one second, and performed the eccentric portion of the contraction over 2-3 seconds. The control group met three times a week for warm-up and stretching exercises, but performed no resistance training. HSPT and SSST performed the same warm-up and stretching exercises as CON.

### 2.4. Outcome Measures

#### 2.4.1. Primary Outcome: Muscle Performance

 Leg press and knee extension 1RM were obtained using Keiser pneumatic resistance training equipment fitted with a420 electronics. As the exercise arm is moved through its range of motion, a piston is driven into a cylinder where it encounters the mechanical resistance of the air pressure in the system. The a420 equipment captured measures of peak power, peak power velocity, and peak power force/torque during the concentric portion of each contraction by sampling the system pressure at 400 Hz and making calculations based on an appropriate algorithm.

The seat of the recumbent leg press and seated knee extension machines were positioned to ensure the hip and knee joint were at 90 to 100 degrees of flexion. The 1RM is defined as the maximum load that can be moved throughout the full range of motion once while maintaining proper form [20]. The 1RM was obtained by progressively increasing resistance until the subject was no longer able to push out one repetition successfully. The Borg scale [21] was used to assist in evaluating when 1RM (combined with perceived maximal effort) was reached. Peak muscle power was obtained at 40%, 50%, 60%, 70%, 80%, and 90% of the 1RM approximately 30 minutes after 1RM testing [10, 11]. Participants were instructed to exert “as fast as possible” at each relative percentage of the 1RM. Three attempts were made at each resistance and the greatest peak power output obtained at each resistance was used in the analysis. The corresponding peak power velocity and peak power force/torque were obtained for each external resistance from 40–90% 1RM. The 1RM was measured biweekly in HSPT and SSST only and relative training intensity was adjusted accordingly to ensure adequate overload during training.

Posttraining muscle performance measures were obtained using loads relative to the initial baseline 1RM to evaluate change in muscle performance variables from baseline to posttraining across a range of external resistances 40–90% 1RM typically encountered in daily tasks. We compared these changes relative to the baseline 1RM because external resistances in the environment are typically fixed and do not increase as you get stronger. This more closely reflects how changes in power and speed apply to “real world” functioning. Sample sizes for the evaluations were: HSPT (*n* = 21), STR (*n* = 23), CON (*n* = 17).

#### 2.4.2. Secondary Outcome: Braking Speed

**Figure 2:**
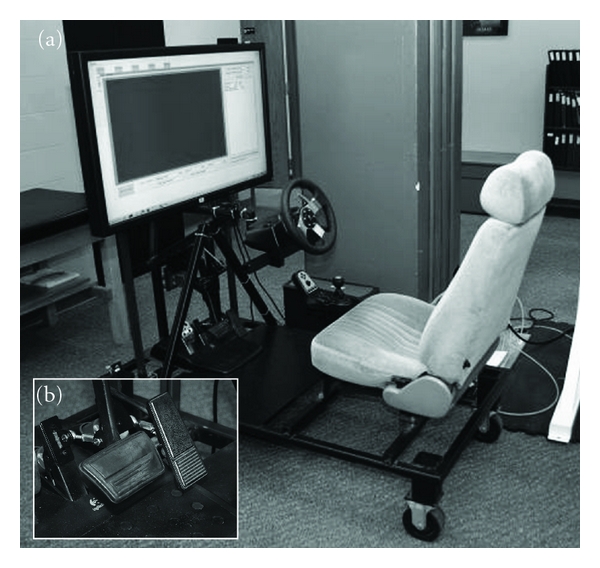
Driving simulator (a) and closeup of accelerator and brake pedal (b) used in the high-speed functional task.

 Braking speed was measured using a driving simulator. The simulator consisted of an adjustable car seat, steering column, and depressable accelerator and brake mounted on a steel frame attached to a computer and wide-screen monitor (Figures 2(a) and 2(b)). The participant was seated with hands on the steering wheel and right foot depressing the accelerator. The participant was instructed to “slam on the brakes” when a visual stimulus changed from green (“go”) to red (“stop”). Pilot work showed that most subjects tend to lift the foot from the accelerator to the brake utilizing the hip, knee, and ankle during emergency braking situations in our laboratory, therefore, we instructed all participants to utilize this strategy instead of simply pivoting the foot between the pedals. In addition, this strategy closely replicated the mechanical movement of the limb during the leg press training. The computer recorded two events (in ms): initial reaction time, or the time for the participant to react to the red light and lift the foot from the accelerator, and the braking speed, the time from movement of the foot off the accelerator and onto the brake. The average of six trials was used in the analysis.

#### 2.4.3. Tertiary Outcomes: Rating of Perceived Exertion and Work

Rating of perceived exertion (RPE) during the training intervention was performed using the Borg scale [21]. The Borg scale consists of a set of numbers that correspond to a specific exertion level. The scale ranges from 6 (no exertion at all) to 20 (maximal exertion), and is commonly used in older adults to determine training intensity [8]. Participants were asked to rate the exertion they felt immediately following each set of leg press and knee extension exercise. The average of the three RPE measures for each leg press and knee extension exercise was calculated to obtain a measure of exertion per exercise session. The RPE per session was then averaged across the total number of exercise training sessions attended during the intervention (12 weeks × 3 visits per week) to provide a measure of average daily RPE. Measures of average daily leg press and knee extension RPE were used in separate analyses.

Work (F × D) was calculated as the mechanical resistance encountered (F) multiplied by the distance the piston traveled into the cylinder (D) during each exercise repetition using the Keiser pneumatic a420 electronics software. Work for each repetition of leg press and knee extension exercise was obtained during each training session and summed for a measure of total work per session. Total work was then averaged across the total number of exercise training sessions attended during the intervention (12 weeks × 3 visits per week) to provide a measure of average daily work. Measures of average daily leg press and knee extension work were used in separate analyses.

### 2.5. Statistical Analyses

Descriptive statistics were run on all variables. Associations among variables of age, sex, body mass index, cognitive function, depression, medications, and falls were evaluated using Pearson's *r*. When significant associations were found, those variables were used as covariates in all analysis of variance (ANOVA) models.

To evaluate baseline differences in subject characteristics, a one-way ANOVA (continuous variables) or chi-square (categorical variables) was run. To evaluate baseline differences among groups in leg press muscle performance (peak power, peak power velocity, and peak power force) at each condition (40–90% 1RM) and braking speed, a univariate ANOVA was run.

To evaluate differences among groups in leg press muscle performance, the change scores in peak power, peak power velocity, and peak power force with training were calculated (posttraining value minus baseline value) at each condition (40–90% 1RM) and a univariate ANOVA was run covarying for the baseline measure. To evaluate differences among groups in braking speed from baseline to posttraining, the change score was calculated (posttraining value minus baseline value) and a univariate ANOVA was run covarying for the baseline measure. If significant group main effects were found, Tukey's HSD test was performed. To determine differences between groups in average daily RPE and work performed during the 12 week intervention, independent samples *t*-tests were performed between HSPT and SSST. Statistical significance for all tests was accepted at *P* < 0.05. Data are reported as means (95% CI).

## 3. Results

### 3.1. Baseline

There were no differences among groups in age, sex, body mass index, depression, cognitive function, number of medications or falls in the past year (see Table 1). Univariate ANOVA showed no group main effects for baseline leg press peak power (all *P* ≥ 0.09), leg press peak power velocity (all *P* ≥ 0.28), or leg press peak power force (all *P* ≥ 0.17) at any condition (40–90% 1RM). Univariate ANOVA showed no group main effects for baseline reaction time (*P* = 0.67) or braking speed (*P* = 0.44). These findings indicate that subject characteristics, muscle performance, and function were similar among all groups at the start of training.

### 3.2. Baseline to after training

#### 3.2.1. Muscle Performance

**Figure 3:**
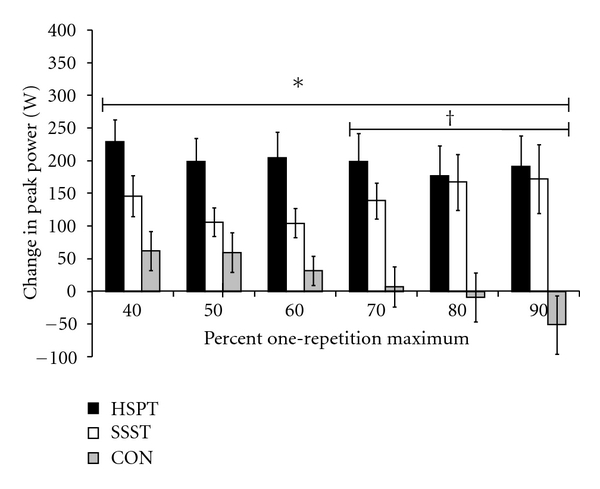
Baseline to posttraining changes in leg press peak power relative to baseline one-repetition maximum (1RM) across a range of external resistances. HSPT = high-speed power training; SSST = slow-speed strength training; CON = control. *HSPT > CON; ^†^SSST > CON.

**Figure 4:**
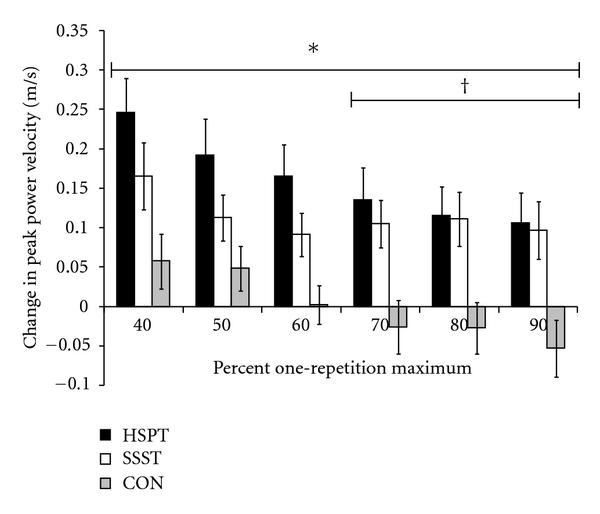
Baseline to posttraining changes in leg press peak power velocity relative to baseline one-repetition maximum (1RM) across a range of external resistances. HSPT = high-speed power training; SSST = slow-speed strength training; CON = control. *HSPT > CON; ^†^SSST > CON.

The changes in leg press peak power, leg press peak power velocity, and leg press peak power force values at each condition (40–90% 1RM) (see Table 2) were compared using a univariate ANOVA. There was a significant group main effects for leg press peak power and peak power velocity at each condition (all *P* ≤ 0.007). Post hoc tests showed that for both measures (peak power and peak power velocity) HSPT was greater than CON across all external resistances (40–90% 1RM; all *P* ≤ 0.02), while SSST was only greater than CON from 70–90% 1RM (all *P* ≤ 0.04) (See Figures 3 and 4). There was no significant group main effect for leg press peak power force at any condition (all *P* ≥ 0.10). These findings indicate that HSPT exerted a broader training effect than SSST when comparing the change in baseline power and speed across a range of typically encountered external resistances.

#### 3.2.2. Braking Speed

Baseline reaction time and braking speed values were 322.9 ms (95% CI: 293.8–352.1 ms) and 221.9 ms (95% CI: 188.5–255.4 ms), respectively, for HSPT; 303.1 ms (95% CI: 270.2–335.8 ms) and 236.1 ms (95% CI: 206.6–265.6 ms), respectively, for SSST; 311.3 ms (95% CI: 269.4–349.8 ms) and 252.0 ms (95% CI: 211.7–292.3 ms), respectively, for CON. There was no significant group main effect for the change in reaction time from the onset of the visual stimulus to the movement of the foot off the accelerator after training (change = −0.79 ms (95% CI: −3.1–1.5 ms) for HSPT, 0.07 ms (95% CI: −1.9–2.1 ms) for SSST, and −1.5 ms (95% CI: −4.4–1.4 ms) for CON; *P* = 0.72). Univariate ANOVA demonstrated a significant group main effect for the change in braking speed with training (*P* = 0.02). The speed at which the lower limb was moved from the accelerator to the brake improved 15.3% in HSPT (change = −3.4 ms (95% CI: −4.8–(−2.0 ms))) and 2.7% in SSST (change = −0.68 ms (95% CI: −3.0–1.7 ms)), but worsened by 2.2% in CON (change = 0.58 ms (95% CI: −1.7–2.8 ms)).

#### 3.2.3. Perceived Exertion/Work

There was no difference between groups in average daily leg press work performed (HSPT: 7235 J (95% CI: 6346–8123 J) versus SSST: 6876 J (95% CI: 5800–7953 J) *P* = 0.59) or knee extension work performed (HSPT: 4002 J [95% CI: 3514–4490 J] versus SSST: 3839 J (95% CI: 3176–4501 J); *P* = 0.68) during the 12-week intervention. There was a difference in average daily RPE between HSPT and SSST (*P* < 0.001) during both leg press and knee extension exercise. Leg press RPE averaged 12.2 (95% CI: 11.5–12.9; “light” to “somewhat hard”) for HSPT and 15.1 (95% CI: 14.3–16.0; “somewhat hard” to “hard”) for SSST training while knee extension RPE averaged 14.6 (95% CI: 13.9–15.2; “somewhat hard” to “hard”) for HSPT and 17.0 (95% CI: 16.2–17.8; “very hard”) for SSST training. These results indicate that despite being exposed to similar workloads during training, HSPT perceived the exercise to be easier.

## 4. Discussion

The major finding from this study was that high-speed power training significantly improved muscle performance and braking speed in older men and women using a driving simulator. High-speed power training and slow-speed strength training both improved power in older men and women; however, high-speed power training also improved the velocity component of power compared to slow-speed strength training and this improvement likely contributed to the improved ability to move the foot quickly from the accelerator to the brake. We believe these findings have significant implications for maintaining safety in older drivers. Because speed is trainable in older adults, the utilization of high-speed movements during resistance training may result in the transfer to functional tasks that require high-speed movements. The benefits to muscle performance obtained at high-speed and low external resistance also occurred without a compromise in muscle strength and at lower perceived exertion compared to slow-speed strength training.

### 4.1. Muscle Performance

We evaluated muscle performance by comparing peak power, peak power velocity, and peak power force across a range of external resistances typically encountered in daily task performance. Because external resistances in the environment are fixed and do not increase as you get stronger, we compared the change in peak power, peak power velocity and peak power force relative to the baseline 1RM, which may more accurately reflect how improvements in muscle performance apply to real-world functioning. We hypothesized that high-speed power training would increase peak power and peak power velocity at the low external training resistances while slow-speed strength training would increase peak power and peak power force at the high external training resistances because of the principle of training specificity. However, HSPT improved peak power and peak power velocity across the entire range of external resistances (40–90% 1RM), while SSST improvements were limited to external loads closest to the training loads (~70–90% 1RM). Although we did not hypothesize that slow-speed strength training would improve peak power velocity, it makes sense that following training (and strength gain) it would be easier to move the same absolute load faster. Still, this did not occur across all external resistances as it did with high-speed power training. Training at 40% 1RM, however, increased peak power and peak power velocity across the entire range of external resistances, demonstrating that high-speed power training exerts a broader training effect than slow-speed strength training.

### 4.2. Braking Speed

A key question was whether improvements in muscle performance with high-speed power training would impact the braking speed functional task. Interestingly, it is not uncommon to find studies where functional tasks do not improve with resistance training or power training. Reviews and meta-analyses [22, 23] demonstrate clearly a very small impact of resistance training on function, with improvements observed mostly with gait-related tasks. Power training studies by Earles et al. [3] and Bean et al. [2] did not find significant changes in function following equipment-based training or training using weighted-vest exercises, respectively. Other power training studies have shown only small changes in sometimes half or fewer of the functional tasks in a battery of tasks [4, 24]. It may be that participants in many resistance training studies are healthy with a greater reserve capacity in their functional abilities. These types of participants are likely closer to their functional threshold, where even large increases in strength or power would result in little or no increases in function. Or it may be that the transfer of the resistance training task did not closely represent the complex movements required for the functional tasks. The transfer of a resistance training task to a functional task is most likely when the muscle activation patterns required for functioning are those that have been repeatedly practiced through the training task [1]. Thus, the optimal transfer of training to function demands specificity between the training task and functional task. Because performance of the braking speed task utilized in this study required considerable movement velocity and a similar movement pattern to the leg press exercise, we anticipated that high-speed training would impact this measure of function to a greater degree than slow-speed resistance training.

Improvement in muscle performance with high-speed power training were closely linked to the improvements in braking speed. When we measured the effect of this relatively simple but explosive movement (moving the foot from the accelerator to the brake) that closely approximated the explosive nature of the exercise (leg press training specifically), we did find positive transfer of training to function. When we examined the leg press power and velocity required for “real world” functional tasks across a range of external resistances, high-speed power training demonstrated greater improvements compared to slow-speed strength training. As a result, the similar braking task may have benefitted from this global improvement in power and speed with high-speed training.

We believe these findings have significant public health implications for older adults who continue to operate motor vehicles. A previous study by Webber and Porter [16] found that power training of the ankle plantar flexors and dorsiflexors at 80% 1RM significantly increased speed of movement from one foot switch to a second foot switch compared to control. Because older drivers are at greater risk for injury-related deaths while driving [15], we believed it was critical to explore this question further by using a driving simulator with an accelerator and brake that more closely represented the equipment and mechanics utilized in a braking maneuver. We calculated that at 60 mph (88 feet per second) with an average deceleration rate of 20 feet per second (coefficient of friction of 0.75), the time to brake a vehicle is 4.4 s (88/20) and 194 feet (half the initial velocity × the time required to stop (0.5 × 88 × 4.4 s)). However, at initial velocity (60 mph), there is a delay in applying the brake due to reaction time to a stimulus and movement time from the accelerator to the brake. In our study, groups had an average of 0.310 s of reaction time plus an additional 0.235 s to move the foot from the accelerator to the brake. Reaction time alone (0.310 s × 88 ft/s) added an additional 27 ft to the distance (194 + 27 = 221 ft). If we calculate a 15.3% improvement in braking time for HSPT (0.198 × 88 = 17 ft) and a 2.2% worsening for CON (0.240 × 88 = 21 ft), differences in stopping distance between the groups will be 242 ft for CON (221 + 21) and 238 feet for HSPT (221+17). Considering that mere inches may be critical to avoid collision-related injury at high speeds, a difference of 4 feet could have significant safety implications.

### 4.3. Perceived Exertion

Finally, older adults performing high-speed power training perceived the exercise to be easier than those performing slow-speed strength training during the 12-week intervention despite both groups performing the same amount of work during the training. These findings could have implications for the retention of older adults in resistance training programs outside the laboratory. Currently, only ~10% of the older adult population participates in resistance training [25]. In addition, for older adults who have previously been involved in resistance training programs maintaining continued participation in this type of exercise has proven difficult. One study reported a 50% decline in the number of older adults participating in resistance training exercise during followup from 12 weeks of facility-based resistance training [26]. Research suggests that moderate intensity exercise is a stronger predictor of whether adults maintain continued participation in exercise than high-intensity exercise [27, 28]; thus, an exercise protocol such as high-speed power training which is perceived as less strenuous could be a part of the strategy to reverse this trend toward reduced participation in resistance training exercise for older adults.

## 5. Conclusions

High-speed power training and traditional slow-speed strength training both improved peak muscle power after 12 weeks of training; however, high-speed power training increased velocity compared to traditional strength training. When examining the power and velocity required for “real world” functional tasks across a range of external resistances, high-speed power training exerted a broader training effect than slow-speed strength training. These benefits occurred without a compromise in muscle strength and at lower perceived exertion (while performing the same amount of work) than when performing slow-speed strength training. Most importantly, high-speed power training improved braking speed using a driving simulator, suggesting that when the explosive nature of the training closely mimicked both the motion and the speed at which the task was performed, there was a positive transfer of training to function. We believe these findings have significant public health implications for our aging population.

## Figures and Tables

**Table 1:** Subject characteristics.

	HSPT (*n* = 25)	SSST (*n* = 25)	CON (*n* = 22)	*P* value
Age (yrs)	70.6 ± 6.7	69.6 ± 8.1	71.1 ± 7.2	0.78
Sex	9 m, 16 f	8 m, 17 f	5 m, 17 f	0.60
BMI	27.3 ± 5.4	29.9 ± 6.9	29.1 ± 6.5	0.32
GDS (0–30)	4.9 ± 4.0	6.1 ± 4.0	6.3 ± 4.5	0.44
MMSE (0–30)	28.5 ± 1.4	28.4 ± 2.1	28.5 ± 1.0	0.95
Medications (no.)	5.4 ± 4.2	4.7 ± 3.0	5.6 ± 3.2	0.63
Falls in past year (no.)	4/25	7/25	3/22	0.40

HSPT = high-speed power training; SSST = slow-speed strength training; CON = control. BMI = body mass index; GDS = Geriatric Depression Scale; MMSE = Mini-Mental State Examination.

**Table 2:** Change in muscle performance across a range of external resistances after 12-weeks of training (using loads relative to baseline 1RM). Data represent mean (95% CI).

Variable		40% 1RM	50% 1RM	60% 1RM	70% 1RM	80% 1RM	90% 1RM
Leg press peak power (W)	HSPT (*n* = 21)	229.0 (159.3–299.3)*	198.7 (124.6–272.8)*	204.9 (123.9–285.8)*	198.5 (108.4–288.6)*	176.9 (80.8–273.1)*	190.9 (92.0–290.0)*
	SSST (*n* = 23)	145.8 (81.1–210.4)	106.2 (61.7–150.7)	104.5 (58.0–150.9)	138.7 (82.0–195.3)*	167.1 (79.1–255.1)*	172.3 63.2–281.4)*
	CON (*n* = 17)	61.9 (−1.8–125.8)	59.7 (−4.4–123.8)	31.8 (−15.5–79.0)	6.9 (−58.9–72.8)	−8.9 (−87.8–70.1)	−51.1 (−145.2–43.0)

Leg press peak power velocity (m/s)	HSPT (*n* = 21)	0.25 (0.15–0.34)*	0.19 (0.10–0.29)*	0.17 (0.08–0.25)*	0.14 (0.05–0.22)*	0.11 (0.04–0.19)*	0.11 (0.03–0.19)*
	SSST (*n* = 23)	0.17 (0.07–0.25)	0.11 (0.05–0.17)	0.09 (0.04–0.15)	0.11 (0.04–0.17)*	0.11 (0.04–0.18)*	0.10 (0.02–0.17)*
	CON (*n* = 17)	0.06 (−0.02–0.13)	0.05 (−0.01–0.11)	0.0 (−0.05–0.05)	−0.02 (−0.09–0.05)	−0.03 (−0.10–0.04)	−0.05 (−0.12–0.02)

Leg press peak power force (N)	HSPT (*n* = 21)	63.3 (37.4–89.1)	57.6 (29.0–86.2)	62.8 (22.5–103.1)	79.0 (40.1–117.2)	78.4 (24.4–132.4)	92.8 (31.3–154.3)
	SSST (*n* = 23)	46.1 (22.8–69.4)	30.1 (8.7–51.6)	30.8 (5.3–56.4)	46.7 (22.8–70.7)	49.2 (21.0–77.4)	51.7 (9.8–93.6)
	CON (*n* = 17)	27.7 (1.5–54.0)	24.9 (−7.0–57.0)	32.3 (−2.1–66.6)	38.6 (3.3–74.0)	38.9 (12.8–65.0)	16.6 (−22.3–55.4)

HSPT = high-speed power training; SSST = slow-speed Strength training; CON = control; 1RM = one-repetition maximum.

*denotes significant difference from CON.
